# Quantitative assessment of left ventricular mechanical dyssynchrony using cine cardiovascular magnetic resonance imaging: inter-study reproducibility

**DOI:** 10.1186/1532-429X-18-S1-P40

**Published:** 2016-01-27

**Authors:** Johannes T Kowallick, Geraint Morton, Pablo Lamata, Roy Jogiya, Shelby Kutty, Gerd Hasenfuss, Joachim Lotz, Amedeo Chiribiri, Eike Nagel, Andreas Schuster

**Affiliations:** 1 grid.7450.60000000123644210Institute for Diagnostic and Interventional Radiology, Georg-August University Goettingen, Goettingen, Germany; 2 grid.13097.3c0000000123226764Division of Imaging Sciences and Biomedical Engineering, The Rayne Institute, St Thomas' Hospital, King's College London, London, UK; 3 grid.418709.30000000404561761Portsmouth Hospitals NHS Trust, Portsmouth, UK; 4 Children's Hospital and Medical Center, University of Nebraska College of Medicine, Omaha, NE USA; 5 grid.7450.60000000123644210Department of Cardiology and Pneumology, Georg-August-University Goettingen, Goettingen, Germany; 6 Division of Cardiovascular Imaging, Goethe University Frankfurt and German Centre for Cardiovascular Research (DZHK, partner site Rhine-Main), Frankfurt, Germany

## Background

We aimed to determine the inter-study reproducibility of left ventricular (LV) mechanical dyssynchrony measures based on standard cardiovascular magnetic resonance (CMR) cine images.

## Methods

Steady state free precession (SSFP) LV short-axis stacks as well as 2-, 3- and 4-chamber views were acquired on the same day at 9:00 (Exam A), 9:30 (Exam B) and 14:00 (Exam C) in 16 healthy volunteers. Circumferential strain systolic dyssynchrony indexes (SDI), area SDI as well as circumferential and radial uniformity ratio estimates (CURE and RURE, respectively) were derived from CMR myocardial feature tracking (CMR-FT) based on the tracking of 3 short-axis planes (basal, mid-ventricular and apical). Furthermore, 4D LV-Analysis based on short-axis stacks and all three longitudinal planes was performed to quantify 4D volume SDI. Exam A and B were compared to assess the inter-study reproducibility. Morning and afternoon scans were compared to study possible diurnal variation.

## Results

CMR-FT derived CURE and RURE as well as 4D LV-Analysis derived volume SDI showed good inter-study reproducibility (coefficient of variation [CoV] 6.4% to 12.9%, intraclass correlation coefficient [ICC] 0.79 to 0.85). Conversely, circumferential strain and area SDI showed higher variability between the repeated measurements (CoV 24.9% to 37.5, ICC 0.67 to 0.76). Averaged CURE and RURE indexes (CURE:RURE_AVG_) showed the lowest inter-study variation (CoV 6.4%) with an ICC of 0.80. Dyssynchrony indexes were not measurably affected by diurnal variation between morning and afternoon scans.

## Conclusions

Derivation of LV mechanical dyssynchrony measures from standard cine images is feasible using CMR-FT and 4D LV-Analysis tools. CURE:RURE_AVG_ and 4D volume SDI showed good inter-study reproducibility. Their clinical value should next be explored in patient groups who potentially benefit from cardiac resynchronization therapy.

